# One Function, Many Faces: Functional Convergence in the Gut Microbiomes of European Marine and Freshwater Fish Unveiled by Bayesian Network Meta-Analysis

**DOI:** 10.3390/ani15192885

**Published:** 2025-10-02

**Authors:** Federico Moroni, Fernando Naya-Català, Genciana Terova, Ricardo Domingo-Bretón, Josep Àlvar Calduch-Giner, Jaume Pérez-Sánchez

**Affiliations:** 1 Fish Nutrigenomics and Integrative Biology Group, Institute of Aquaculture Torre de la Sal (IATS, CSIC), 12595 Castellón, Spain; fernando.naya@iats.csic.es (F.N.-C.); ricardo.domingo@csic.es (R.D.-B.); j.calduch@csic.es (J.À.C.-G.); 2 Department of Biotechnology and Life Sciences, University of Insubria, 21100 Varese, Italy; genciana.terova@uninsubria.it

**Keywords:** fish gut microbiome, core microbiota, host genetics, dietary intervention, taxonomical variability, microbiota biomarkers, Bayesian network, bacterial functions, European sea bass, Rainbow trout

## Abstract

**Simple Summary:**

To address the challenge of assessing how diet, environment, and genetics shape fish gut microbiota of different species, in this work we integrated taxonomic, functional, and network-based approaches. Analyzing sea bass, trout, and sea bream, we identified a conserved core microbiota with central hierarchical and functional roles participating significantly in key metabolic pathways. Despite taxonomic variability, core-associated clusters showed strong positive interactions and specific biomarkers across ecological contexts. These results provide a robust framework for monitoring intestinal health, defining welfare indicators, and supporting sustainable aquaculture through targeted strategies.

**Abstract:**

Intestinal microbiota populations are constantly shaped by both intrinsic and extrinsic factors, including diet, environment, and host genetics. As a result, understanding how to assess, monitor, and exploit microbiome–host interplay remains an active area of investigation, especially in aquaculture. In this study, we analyzed the taxonomic structure and functional potential of the intestinal microbiota of European sea bass and rainbow trout, incorporating gilthead sea bream as a final reference. The results showed that the identified core microbiota (40 taxa for sea bass and 20 for trout) held a central role in community organization, despite taxonomic variability, and exhibited a predominant number of positive connections (>60% for both species) with the rest of the microbial community in a Bayesian network. From a functional perspective, core-associated bacterial clusters (75% for sea bass and 81% for sea bream) accounted for the majority of predicted metabolic pathways (core contribution: >75% in sea bass and >87% in trout), particularly those involved in carbohydrate, amino acid, and vitamin metabolism. Comparative analysis across ecological phenotypes highlighted distinct microbial biomarkers, with genera such as *Vibrio*, *Pseudoalteromonas*, and *Paracoccus* enriched in saltwater species (*Dicentrarchus labrax* and *Sparus aurata*) and *Mycoplasma* and *Clostridium* in freshwater (*Oncorhynchus mykiss*). Overall, this study underscores the value of integrating taxonomic, functional, and network-based approaches as practical tools to monitor intestinal health status, assess welfare, and guide the development of more sustainable production strategies in aquaculture.

## 1. Introduction

All living organisms are exposed to a wide range of microbial populations, whose colonization and transmission within the host’s body niches result from a combination of environmental and parental effects. The environmental effects primarily lie to a particular habitat, social interactions among relatives or closely associated groups, as well as dietary preferences or hygiene practices [1]. By contrast, parental pathways rely primarily on vertical transmission or on host genetic traits that actively influence the composition of the associated microbiota [2]. In that sense, studies in humans [3,4], primates [5], and livestock animals (e.g., cattle, swine, and poultry) have described a strong influence of host genetics on the microbiota of various mucosal surfaces [6,7,8]. Aquaculture farmed fish are not an exception, and the influence of genetic traits upon the fish microbiota remains equally decisive. This notion is first supported by the observation that the skin and intestine microbiota of both freshwater and seawater fish does not exactly mirror the microbiota characteristic of their surroundings, suggesting that host-specific selective pressures, including immune defenses, nutrient availability, physicochemical conditions, and microbe–microbe interactions, outweigh environmental influences [9,10,11,12]. Further confirmation of this hypothesis comes from studies conducted within a common garden system, in which different families with different genetics were placed in the same experimental conditions. Gilthead sea bream families selected for fast growth exhibited a less heterogeneous but more plastic gut microbiota, in which small changes in bacterial composition resulted in larger changes in metabolic capacity, allowing selected fish to better cope with changes in diet composition and bacterial challenges [13,14]. Additionally, the genetic background also determined how the successions of gut microbiota populations are driven across season [15,16] as well as how it is differentially modulated by a vast array of feed additives, including phytogenics, organic acids, and probiotics [17,18].

The host–microbiome system represents a network of synergies and functional interactions that develop and consolidate over time, reflecting co-evolutionary processes in which the microorganisms inhabiting the host or its surroundings constitute the same eco-physiological unit, the holobiont [19,20,21]. In that sense, an increasing number of studies with germ-free models have shown that larvae, juvenile, and adult fish often fail to develop a stable microbial population [22,23,24]. The number of microorganisms that can establish a positive equilibrium with the host through mutualistic and commensal relationships far exceed those involved in opportunistic or parasitic interactions. As a result, host-associated microbiota communities, which actively regulate a wide range of physiological processes in all living organisms, including fish, are highly variable and contribute to improved growth performance, nutrient digestion and absorption, immune responses, disease resistance, and stress resilience and tolerance [25,26,27,28]. This occurs through the microbial production of locally and systemically active compounds, such as short-chain fatty acids, vitamins, enzymes, and hormone precursors [17,29,30], which can reach the central nervous system with notable implications for behavior and neurological function [31,32,33,34]. The significance of these interactions is increasingly recognized, and microbiota profiling, mostly at the gut level, is emerging as a new criterion for assessing the health and welfare of farmed fish under different feeding regimes [16,35], elevated temperatures [36,37], and crowding-related stress [38]. In parallel, the skin microbiota is also gaining attention as a complementary indicator, showing high responsiveness to high stocking densities and limited oxygen availability, becoming a much less invasive approach for assessing fish welfare and wellbeing [39,40]. However, our current understanding of fish microbiota regulation remains limited in comparison to what is known in humans and other animal models [41]. Most of the fish studies are in fact largely descriptive and focused uniquely on the taxonomical characterization of the microbial profile, leaving its functional role, the causal relationships within the network, and its coordination with host physiology mostly underdeveloped [22,24]. For this reason, there is a pressing need for more comprehensive and standardized approaches, both in experimental design and data interpretation, together with the application of machine learning and multi-omics integration techniques to uncover species-specific patterns as well as conserved microbial traits across several farmed fish [42,43]. In this way, an effective strategy for advancing in microbiota research is by revisiting the core microbiota, defined as the subset of microbial taxa that is consistently present, typically stable, and abundant across individuals of a species [44]. Indeed, identifying the most influential taxa with a key ecological and functional role within the host–microbe system could help to address the challenging variability and functional redundancy that characterizes microbial communities [45,46,47].

Despite all the above findings and the rapid surge in microbiome research, progress is hindered by inconsistencies in methodological standardization, the prevalence of descriptive studies lacking integration and contextualization, and the absence of a universally accepted core functional microbiota. Together, these limitations make it difficult to identify common traits and establish reliable references across research fields, including humans, livestock, and aquatic organisms. Accordingly, the present study represents an exploratory attempt to investigate both the specificity and overlaps of the intestinal core microbiota focusing on two of the most important marine and freshwater farmed species for the European aquaculture and Mediterranean basin: European sea bass (*Dicentrarchus labrax*) and rainbow trout (*Oncorhynchus mykiss*). To reinforce this approach, a third commercially important species, the gilthead sea bream (*Sparus aurata*), was used as a reference dataset derived from various dietary interventions applied to the same batch of fish [48]. To this end, this first integrative and novel analysis went beyond conventional taxonomic profiling by incorporating functional and hierarchical interpretations through Bayesian network (BN) analysis. Although focusing on a limited number of species, this strategy provides a proof of concept for assessing how alternative protein sources, host genetics, and freshwater/seawater environment shape gut-associated bacterial communities and paves the way for future studies with a wider range of farmed species. By considering these factors and using the core microbiota as the analytical foundation, this meta-analysis aimed not only to identify species- and environment-specific bacterial biomarkers but also to reveal common microbiota traits that could serve as universal indicators for evaluating and certifying welfare status in farmed fish.

## 2. Materials and Methods

### 2.1. Experimental Microbial Datasets

The present multi-species meta-analysis was conducted using a total of seven intestinal microbiota datasets: four for sea bass, defined as Tr.SB1 [49], Tr.SB2-3 [50,51], and Tr.SB4 [18], and three for trout, defined as Tr.T1-2 [52,53] and Tr.T3 [54]. The starting data matrix reflects the changes in the gut microbiota due to the partial replacement of fishmeal (FM) and fish oil (FO) and the use of alternative feed ingredients and additives, such as animal by-products, insect meal, phytogenics, organic acids, and pre- and probiotics. Detailed dietary information is reported in Table S1. In addition, sea bass specimens used in Tr.SB3 and 4 belonged to the same selective program for high growth performance. Similarly, although not selected for growth, the trout of Tr.T2 and 3 shared a common genetic background. The animals used in the remaining trials were derived from distinct genetic batches. In all trials, microbiota analyses were conducted using high-throughput sequencing targeting the V3 and V4 hypervariable regions of the 16S rRNA gene following the Illumina protocol “16S Metagenomic Sequencing Library Preparation” for the Illumina MiSeq System. The entire microbiota profiles of all trials for each species were then merged to obtain two different multi-trial datasets, which rendered a total of 737 and 444 Operational Taxonomic Units (OTUs) for sea bass and trout, respectively. Sample depth was normalized by total sum scaling and then made proportional to the total sequencing depth [55], and microbiota taxonomy was updated according to the SILVA v138.1 database. An additional microbiota dataset from a previously published gilthead sea bream meta-analysis [48], which included multiple dietary interventions applied to a genetically homogeneous batch of fish, was used in the present study for a final three-species comparison.

### 2.2. The Bayesian Network Construction

The microbiota raw counts, taken from the sea bass and trout datasets, were used to perform a functional meta-analysis using a BN approach. The construction of the networks, one for each fish species, was built using the SAMBA tool following the settings reported elsewhere [48,56]. In brief, taxa with normalized zero total counts were filtered from the datasets before the execution of the network. Zero inflated Negative Binomial (ZINB) distribution was used to fit the models, while the strength of each connection (edge) was calculated using the Bayesian information criterion (BIC), ensuring an optimal balance between network complexity and explanatory power, preventing overfitting, and mutual information (MI) criterion, filtering weak or spurious associations, fixing the thresholds at 0 and 0.05, respectively. The condition “experiment”, which summarizes the differences between the trials, was used as a driver variable in the BN constructions. A clustering analysis was then performed on the resulting networks using the Leiden community detection method to identify groups of densely connected nodes within the microbiota structure [57].

### 2.3. Functional Inferred Metagenome Profile

The functional contribution of the microbiota profiles was inferred from 16S rRNA data using the PICRUSt2 protocol and the Kyoto Encyclopedia of Genes and Genomes database (KEGG) [58] as a reference, implemented in the SAMBA platform [56]. The bacterial metabolic pathways analysis was carried out starting from the nodes (OTUs) which compose the two Bayesian networks, and the cluster organization was obtained.

### 2.4. Statistical Analysis

Before the analysis, normality of the data was verified by the Shapiro–Wilk test. Differences in the relative abundance of the intestinal microbiota profiles for each fish species (sea bass and trout) at the phylum level were then analyzed using the Kruskal–Wallis test followed by Dunn’s post-test for multiple comparisons. The significance threshold was set to *p* < 0.05. Bacterial profiles were also investigated by partial least squares discriminant analysis (PLS-DA) using EZinfo v3.0 (Umetrics, Umeå, Sweden). The outlier’s identification was performed using Hotelling’s T2 statistic, setting a 95% confidence limit for T2. The quality of the PLS-DA models was confirmed by the parameters R2Y (cum) and Q2 (cum), and the validation tests were performed with the Bioconductor R package ropls v3.21 [59], consisting of 500 random permutations. The contribution of different OTUs to group separation was determined by the variable importance in projection (VIP) using a VIP threshold of ≥1. The identified VIPs were used for the hierarchical clustering using the R package ggplot2 v4.0.0. In the multi-species model, loadings values were analyzed to identify the group-specific OTUs. To do so, a threshold based on the statistical distribution of the loading was applied. OTUs with loading values greater or less than ±1.96 standard deviations from the mean (95% confidence interval in a normal distribution) were considered significantly associated with one of the groups. For the evaluation of functional inferred metagenomic profiles, raw KEGG pathway data was then normalized within each fish species dataset and analyzed using the Kruskal–Wallis test with a significance threshold of *p* < 0.05.

## 3. Results

### 3.1. Main Driving Factors in Microbiota Profiling

**Figure 1:**
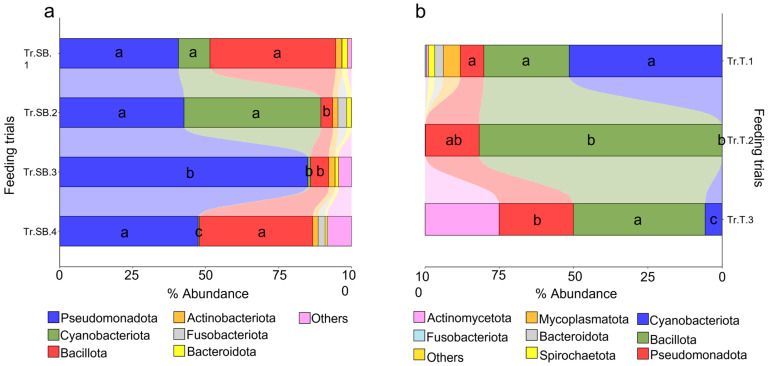
Stacked bar charts representing the relative abundance of bacterial phyla for each farmed species considered. (**a**) Feeding trials for sea bass (Tr.SB.1-4). (**b**) Feeding trials for trout (Tr.T1-3). Different letters indicate statistically significant differences (Kruskal–Wallis test, *p* < 0.05) in the three main phyla between the feeding trials.

**Figure 2:**
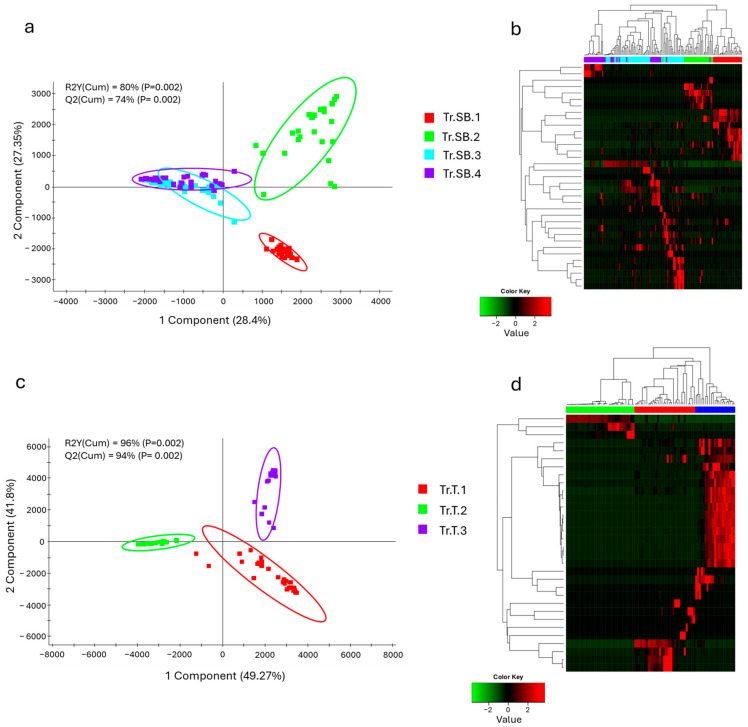
Two-dimensional representation of sample distribution of samples along the first two components of the partial least squares discriminant analysis (PLS-DA) model, driving the separation between different feeding trials along with the heatmap showing the hierarchical clustering of VIPs (VIP value > 1). (**a**,**b**) PLS-DA and clustering heatmap for sea bass. (**c**,**d**) PLS-DA and clustering heatmap for trout. Fish belonging to Tr.SB.3-4 for sea bass and Tr.T.2-3 for trout shared the same genetic background.

With the aim of identifying the differences between the different trials considered, the microbiota profiles were analyzed starting from the taxa distribution. For each species, differences between feeding groups were already evident at the phylum level (Figure 1a). The results highlighted that in sea bass, the three main important phyla were Pseudomonadota, Cyanobacteriota, and Bacillota, which together account for almost 80% of the total abundance, although with a high degree of variability across all trials. Due to their abundance, these taxa also represented the most significant changes between trials. Pseudomonadota exhibited a conserved abundance pattern, with the only exception of Tr.SB.3, whereas Cyanobacteriota showed a more discontinuous distribution passing from the 10% in Tr.SB.1 and almost 50% in Tr.SB.2 to being practically absent in the rest of the trials. The Bacillota phylum instead highlighted a similar abundance value in trials Tr.SB.1 and Tr.SB.4 that diverged from that found in trials Tr.SB.2 and Tr.SB.3. Thus, the validated (Figure S1a) PLS-DA approach depicted a different trial grouping (Figure 2a), where the two first components, describing 80% of the observed variance (R2Y (cum), *p* < 0.02) and the 74% of the predicted variance (Q2 (cum), *p* < 0.02), clustered together Tr.SB.3 and Tr.SB.4, while trials Tr.SB.1 and Tr.SB.2 formed separated groups. As reported in Figure 2b, a total of 35 OTUs with VIPs > 1 contributed to the separation of the samples in the hierarchical clustering according to the three main blocks identified in the PLS-DA model (Tr.SB.1, Tr.SB.2, and Tr.SB.3+4), combining the two trials with fish belonging to the same selective program.

Phylum distribution in trout microbiota profiles appeared more heterogeneous (Figure 1b). Cyanobacteriota, Bacillota, and Pseudomonadota showed the same relative importance to that observed in sea bass but highlighting a different hierarchical order. Cyanobacteriota dominated the microbial profile in Tr.T.1 but dropped to very low values in the other two trials. In contrasts, Bacillota represented almost 80% of the total abundance of Tr.T.2, while its presence declined to 25% and 45% in Tr.T.1 and 3, respectively. Lastly, unlike the results obtained in sea bass, Pseudomonadota accounted for a smaller fraction and relatively stable portion of the microbiota profile across all trout trials. The distribution patterns observed at the phylum level were supported by the statistically validated multivariate analysis (Figure S1b), where the three trout trials were clearly separated along the first two components of the PLS-DA model (Figure 2c), which together account for 96% of the observed variance (R2Y (cum), *p* < 0.02) and 94% of the predicted variance (Q2 (cum), *p* < 0.02). In this case, the discrimination of the groups in the hierarchical clustering (Figure 2d) was driven by a total of 32 bacteria having a VIP value > 1, which separated the three trials regardless of the genetic background.

### 3.2. Core Microbiota Contribution Within the Population

**Figure 3:**
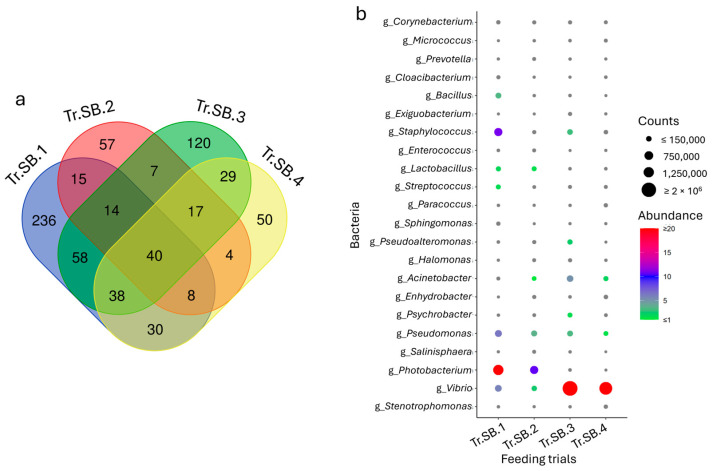
(**a**) Venn diagram reporting unique and shared taxa considering the sea bass intestinal microbiota datasets of the four feeding trials. (**b**) Dot plot representation of the most abundant fraction (taxa average abundance > 0.1%).

**Figure 4:**
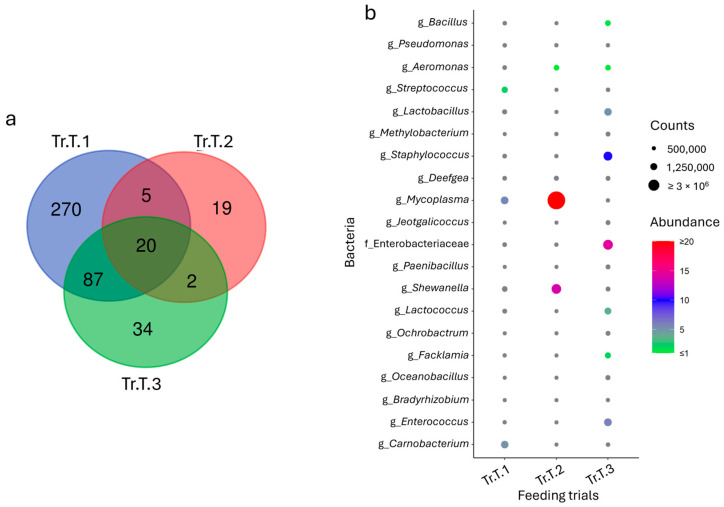
(**a**) Venn diagram reporting unique and shared taxa considering the trout intestinal microbiota datasets of the three feeding trials. (**b**) Dot plot representation of the core microbial fraction.

To identify the common fraction of the microbiota across the trials, the core microbiota was calculated for each species. Despite the differences already highlighted in Section 3.1, the analysis of data highlighted a total of 40 and 20 taxa for sea bass and trout, respectively, which were consistently shared and abundant in all the trials (Figure 3a and Figure 4a). These population fractions, identified almost entirely at the genus level, mirrored the grouping obtained in the multivariate analysis, especially for sea bass, where trials Tr.SB.3 and Tr.SB.4 appeared more similar to each other than to the remaining trials (Figure 3b). Relevant in this context are the bacteria belonging to the genera *Vibrio*, *Bacillus*, *Micrococcus*, *Lactobacillus*, and *Streptococcus*, which, with their relative abundance higher than 0.5% in at least one feeding trial, may also play a key role in functional dynamics. In contrast, the core microbiota distribution in trout remained variable across trials (Figure 4b). For example, *Mycoplasma* and *Shewanella* (which together represent more than 90% of the profile) were mainly associated with Tr.T.2, while *Staphylococcus* (≈10%), *Enterococcus* (≈6%), and *Lactobacillus* (5%) were more abundant in Tr.T.3. Tr.T.1, however, displayed the most homogeneous distribution even in the core, with *Mycoplasma*, *Carnobacterium*, and *Streptococcus* as the most represented genera, accounting for approximately 2% to 5% of the total abundance.

### 3.3. Hierarchical and Functional Role of Core Microbiota in Bayesian Network Models

**Figure 5:**
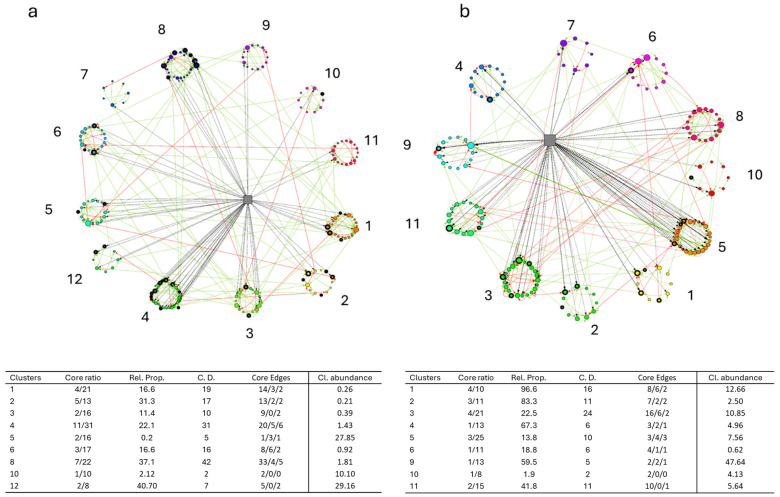
Bayesian networks representing sea bass (**a**) and trout (**b**) models. Circles represent bacterial taxa and gray squares represent the variable “Experiment”. The core microbiota taxa are indicated by the black edges of the circles. Green and red arrows represent positive and negative edges, while the dashed black arrows indicate edges with the variable “Experiment”. The tables report the clusters which include core taxa and indicate the number of core bacteria compared to the total number of bacteria in the cluster and the proportion (Core ratio and Rel. Prop.); the number of connections of the core bacteria defined as centrality degree (C. D); the number of positive and negative edges and those involving the variable “Experiment” (Core edges); and relative abundance of the whole cluster (Cl. abundance).

The construction of the BN enabled a more detailed analysis of the intestinal microbial profiles, revealing the real distribution and hierarchical role of the identified core microbiota of sea bass and trout, respectively (Figure 5a, b). The results showed that 9 out of 12 clusters in sea bass and 11 in trout included at least one core taxa. Particularly noteworthy is the number of connections (edges) established by these bacteria with other members of their cluster and/or with the broader microbial community. For example, clusters 1, 2, 4, 6, and 8 in sea bass and clusters 1, 2, 3, and 11 in trout exhibited a predominance of positive over negative connections. This suggests that cooperative and mutualistic relationships prevail over competitive interactions within these microbial communities. Additionally, these clusters were also connected with the variable “experiment”, indicating that experimental conditions played a role in modulating the composition and structure of the core microbiota. Further information on the networks, including centrality degree, betweenness, and closeness metrics, are reported in Table S3.

**Figure 6:**
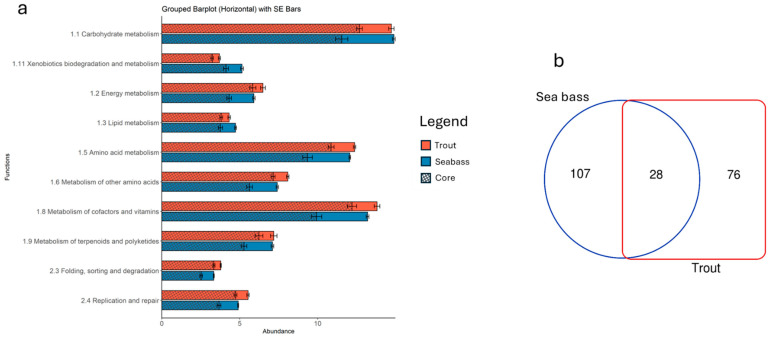
(**a**) Functional representation of the main metabolic pathways (relative abundance > 3%) of KEGG annotations for sea bass and trout reporting both the total and core contributions. (**b**) Venn diagram of the total number of shared functions between sea bass and trout.

Regarding the functional analysis performed on the two BNs, although inference from 16S rRNA data may introduce biases for poorly characterized taxa, the present findings indicated a highly conserved expression of metabolic routes exhibited by the gut microbial communities of the two farmed fish studied. Figure 6a displays the top 10 metabolic pathways (each accounting for more than 3% of total abundance), organized according to the second grouping level of the KEGG hierarchy and shared between trout and sea bass. Among the most represented functions, those related to carbohydrate metabolism, amino acid metabolism, cofactors, vitamins, and energy metabolism are noteworthy. Despite these similarities, the statistical analysis revealed significant differences in nearly all pathways, both at the highest (level 3) or lowest (level 2) resolution level of the rankings defined in the KEGG database (Table S2). While this might seem contradictory, it actually reflects a low dispersion of values, which in turn amplifies the significance of the observed differences in the inferred functional profiles of the two microbiomes, even though both maintain a strong tendency toward functional redundancy. This same trend was also observed in the distribution of inferred functions associated with the core microbiota. Specifically, the calculation of metabolic contributions from clusters containing core taxa revealed marked differences between the two farmed fish species, despite an overall, though less pronounced, homogeneity in distribution. Notably, these values demonstrate that the core microbiota, along with its closely connected members, contributes almost the maximum functional expression observed across the entire microbial community for each function analyzed.

The functional redundancy exhibited at the pathway level, however, does not correspond taxonomically to the bacterial taxa responsible for those inferred functions. As shown in Figure 6b, from the total number of bacteria involved in this analysis (211), only a small fraction (approximately 13%) was shared between the two microbiomes. This limited overlap aligns with previous observations at both the phylum level and at the composition of the core microbiota. These findings further underscore the distinct distribution of key taxa within the two intestinal environments, which nonetheless converge at the functional level.

### 3.4. Multi-Species Phenotyping of Microbiota Markers

**Figure 7:**
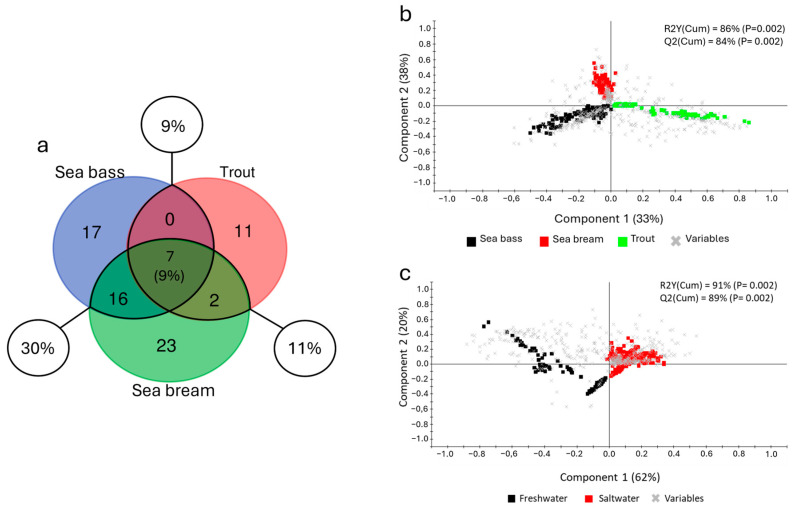
(**a**) Venn diagram of the shared taxa between the three different core profiles identified, showing the total proportion of shared taxa (9%) and the three couplings between species: sea bass–trout (9%); trout–sea bream (11%); and sea bass–sea bream (30%). Two-dimensional representation of the distribution of samples between the first two components of the partial least squares discriminant analysis (PLS-DA) model driving the separation of the three different fish species (sea bass, sea bream, and trout) (**b**) and the two ecological groupings (freshwater and saltwater) (**c**). Squares represent samples while crosses represent single taxa (variables).

Apart from the feed composition, which largely represents the main factor affecting the intestinal microbiota composition, the present study also aimed to investigate another extrinsic variable, identified as the environment in which each farmed fish species typically resides. This was made by introducing a dataset from a meta-analysis conducted on sea bream, which revealed greater similarities between the two seawater species (sea bass and sea bream) than with the freshwater species, trout (Figure 7). Starting from the core microbiota, the sea bass and trout profile were compared with the sea bream core previously described [48] using a three-way evaluation (Venn diagram). Given that the sea bream samples included in the meta-analysis shared a common genetic background, their microbiota compositions were highly similar. Hence, to ensure a balanced and comparable analysis across species while maintaining an equivalent level of representativeness, we considered the filtered sea bream core microbiota, which included 48 taxa. As shown in Figure 7a, only seven genera (9% of the total), including *Bacillus*, *Pseudomonas*, and *Lactobacillus*, were shared across all species. However, the number of shared bacteria increased significantly when only the two seawater species were considered (sea bass–sea bream 30%), while instead they remained at a lower and almost equal proportion when compared with trout (sea bass: 9%; sea bream: 11%). These results were further corroborated by the discriminant analyses. In fact, the initial PLS-DA model showed a dispersion of the data much more marked in the trout dataset along the first component axis, which accounts for 33% of the total explained variance. In contrast, the differences between sea bass and sea bream, though present, were only distinguishable along the second component, explained the 38% of variance (Figure 7b). A clearer and more distinct separation emerged when considering the two environmental phenotypes (freshwater and saltwater) in a single multivariate analysis. Even though both of the PLS-DA model results were significantly validated (Figure S2a,b), the second approach yielded more robust results, increasing both the observed variance (R2Y (cum), *p* < 0.02) from 86 to 91% as well as the predicted variance (Q2 (cum), *p* < 0.02) from 84 to 89% (Figure 7c).

**Figure 8:**
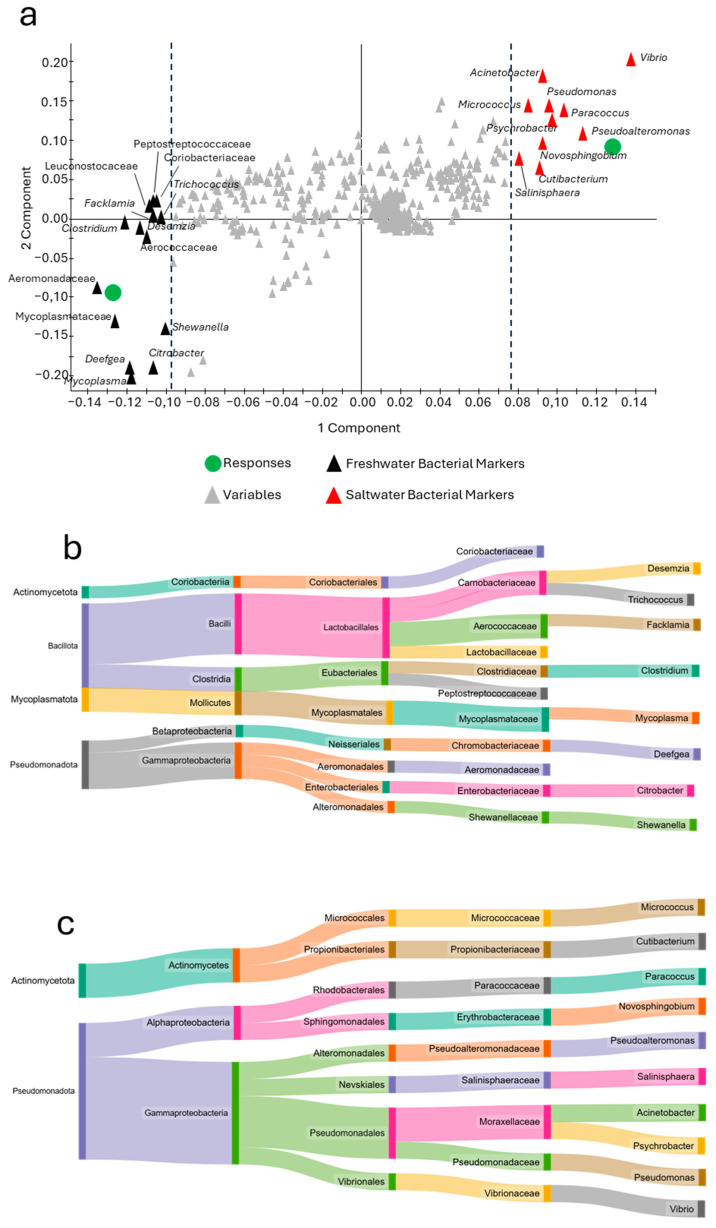
Loading plot of the ecological grouping in the PLSDA model shown in Figure 7c reporting the main taxa markers associated with each group (**a**). Sankey diagrams reporting the list of bacterial markers identified for freshwater (**b**) and saltwater (**c**).

A more in-depth analysis of this model using the loading plot evaluation (Figure 8a) revealed a clear grouping of the bacteria according to the two ecological profiles. The results showed that the freshwater phenotype was enriched in the genera *Deefgea*, *Mycoplasma*, and *Clostridium*, together with taxa belonging to the Aeromonadaceae and Mycoplasmataceae families, exhibiting a generally more biodiverse marker profile, including four phyla and six classes (Figure 8b). In contrast, the seawater phenotype highlighted more taxonomically conservative indicators. Figure 8c, in fact, reveals that all 10 taxa identified are included in only two phyla (Actinomycetota and Pseudomonadota), with the genera *Vibrio*, *Pseudoalternomonas*, *Paracoccus*, *Psychrobacter*, and *Pseudomonas* being particularly prominent, showing a strong association with the marine water phenotype.

## 4. Discussion

The study of host-associated microbiota has emerged as a cornerstone of modern ecological and biomedical research due to its dual value in both descriptive profiling and predictive diagnostics [26,60]. However, despite the significant and continuous growth of microbiome-focused research, the complex and highly individualized nature of environment–host–microbiota interactions continue to hinder a comprehensive understanding of the general and specific mechanisms that govern these networks in both organisms and ecosystems [61,62]. In this context, the identification of consistent and biologically relevant microbial taxa, which contribute most to host–microbe interactions, is essential for developing a reliable set of microbiome-derived biomarkers with both functional and hierarchical significance. This research perspective is especially valuable, as it facilitates the integration of multiple layers of data (taxonomic, functional, and phenotypic) starting from cost-effective and easily accessible sequencing approaches. In that sense, the advent of rapid on-site technologies such as 16S rRNA gene sequencing, supported by increasingly standardized bioinformatic pipelines and predictive tools, are becoming highly accessible and scalable [43,63,64]. These advancements are reinforcing the use of microbiota profiling as a strategic instrument for promoting sustainable aquaculture production, even under resource-limited conditions [56,65,66].

The great taxonomic variability in microbiota is the result of multiple factors that influence both the external and internal bacterial communities associated with the host. In aquaculture and other ecosystems, the principal elements shaping microbiota populations are the environment, host genetics, and dietary habits [27,67,68]. Even within a single host species, these variables can induce significant shifts in microbiota composition and function, sometimes observable even at high taxonomic ranks [69]. Accordingly, in the present study, the analysis of sea bass and trout trials revealed notable changes even at the phylum level. In fact, Pseudomonadota, Cyanobacteriota, and Bacillota (the three dominant phyla) exhibited variable relative abundances depending on the feeding regime. In agreement with this, numerous studies have analyzed how FM and FO replacers affect the distribution of intestinal taxa in various farmed fish, including salmonids, shrimp, tilapia, and other freshwater and marine organisms [70,71]. In particular, in trout, both Rimoldi et al. (2021) and Pérez-Pascual et al. (2021) have reported positive effects of insect meal inclusion on the abundance of the Bacillota phylum (formerly Firmicutes), particularly the Bacilli class, alongside a reduction in the relative abundance of Pseudomonadota (formerly Proteobacteria) [72,73]. In addition to diet, host genetics is also widely recognized as a key factor influencing gut microbiota composition [12]. This assumption is also supported by the present study, in which comparisons are made within and between sea bass and trout. For instance, despite the notable heterogeneity in phylum-level distribution among sea bass trials, the supervised multivariate PLS-DA analysis revealed a distinct clustering of fish lines selected for growth performance, whereas individuals from non-selected genetic backgrounds displayed greater dispersion. These results suggest that genetic selection for traits like enhanced growth might co-select for a particular gut microbial consortium, possibly due to associated physiological traits such as digestion rate or feed conversion efficiency [13]. A similar pattern was also documented for gilthead sea bream. Thus, Naya-Català and colleagues (2022) reported that genetically selected fish showed a less biodiverse and more conserved microbial profile but at the same time a greater metabolic capacity capable of adapting to dietary changes without requiring major shifts in taxonomic composition [16]. This functional plasticity reinforces the concept that selective breeding not only affects host traits but also indirectly modulates the potential of the gut microbiome, selecting those low-abundant taxa that cause high variability and increasing the relative importance of already abundant taxa both hierarchically and functionally, co-regulating and optimizing the physiological performance [17,74]. The link between host genetics and microbiota composition is well established in other areas of animal production, including cattle, swine, and poultry, as well as in mice and humans [2,6,7]. However, it must be noted that our meta-analysis of trout microbiota profiles of fish with similar genetic backgrounds still displayed notable inter-individual variability, resulting in divergent microbiota compositions. Such variability demonstrates how, in the absence of a stronger variable, such as genetic selection, stochastic microbial colonization, environmental factors, and dietary changes contribute to increasing differences between communities. These findings align with previous results in gilthead sea bream, where a clear separation of microbial communities due to different feeding sources was observed despite the shared genetic background [48].

Despite the differences found both at higher and lower taxonomic levels, the analysis of the core microbiota represents a valuable strategy for evaluating and monitoring the impact of various extrinsic and intrinsic factors on microbial communities [75]. This subset of taxa has been shown to serve as a reliable baseline for identifying common traits and general similarities across different trials without requiring a complex evaluation of the entire bacterial population. For instance, Moroni et al. (2025) investigated the effects of dietary FM and FO replacers on the intestinal microbiota of gilthead sea bream and found that changes in the core microbiota closely mirrored those observed in the overall microbial community [48]. The authors described how the changes in the core microbiota profile mirrored the changes and the similitude obtained by analyzing the entire population, allowing for a more precise evaluation based on markers that are consistently found across individuals and regardless of different conditions. Due to its stability and relative abundance, in fact, the core microbiota constitutes a central point both from a hierarchical and functional point of view in the host-associated microbiome and in the physiological interactions within the holobiont system (microbiome and host association) [60,76,77]. The core microbiota profile of sea bass, indeed, showed a relatively high similarity between the two feeding trials performed using genetic selected animals. This convergence, driven by genera like *Vibrio*, above all, but also *Lactobacillus*, *Streptococcus*, and *Photobacterium*, confirmed what already discussed in the multivariate analysis reinforcing the advantages in using core microbiota. However, while this approach was helpful for identifying common patterns across trials and conditions, it only focuses on taxonomy and does not provide information about the functional roles of the microbial community [44,78]. For this reason, our investigation also deepens in the network construction using a Bayesian approach. As expected, in the BN of both fish species, the core microbiota appeared to be widely spread and distributed in the population, likely due to its generalist nature and its ability to exploit a wide range of resources and substrates [79,80]. Furthermore, the presence of the core microbiota in the numerous clusters obtained also suggests a hierarchical role, which results in the modulation of cluster-specific metabolic processes or involves the entire intestinal microbial population. This function in the community is also manifested in the high number of edges in which the core is responsible both as a father, where it therefore manages the abundance of other taxa, and as a child, in which instead its presence is subordinated to the dynamics of other taxa.

Microorganisms form complex networks within and between species across spatial and temporal scales using cooperative and antagonistic interactions [81]. Positive associations often arise from processes like cross-feeding and mutualistic relationships, also called syntrophism, characterized by complementary metabolism and functions that create favorable niches for one another. In contrast, negative connections may result from competition, amensalism, or predation, in which species inhibit each other’s growth and survival [82,83]. However, the interpretation of this pattern is not straightforward, even more so when using metagenomic techniques. The use of computational analysis, therefore, such as the Bayesian network built in this study, represents a promising parallel advance to detect relationships in such complex bacterial associations [84]. In the present condition, our results showed that for both fish species analyzed, most of the connections identified within the networks, and especially those involving the core microbiota, are positive, which suggest a strong synergy and cooperation between taxa rather than a real biological and spatial competition. Similar findings were also reported by Kokou et al. (2019) [85]. Investigating the interactions within microbiota using different techniques, including microbiological and computational analysis, the authors described the prevalence of positive or weakly competitive interactions between the core microbiota, pointing to a different substrate utilization and thus the complementary ecological role of the taxa belonging to this group [85]. According to this positive influence of the core microbiota, the metabolic pathway analysis also showed that, for each function examined, the combined contribution of the core taxa and their closely related cluster members accounted for nearly the entire functional value of the whole microbial community. Notably, this aspect of dominance was found in both species analyzed. Indeed, even considering the two different profiles, at the metabolic level, the two compartments behave in the same way, exhibiting a strong functional redundancy and comparable ratios. Furthermore, in addition to the similarity in the expression levels of the physiological functions, these results also underline the strong influence of microbiota populations on digestive processes, and in particular with the management of the principal biomolecules. Higher values were in fact achieved in carbohydrate, amino acid, and energy metabolism, together with the production and synthesis of cofactors and vitamins, which represent a strong connection point between host and microbiota physiology [31,86,87]. In any case, the functional convergence reported in this study is in line with the findings described in gilthead sea bream [48]. Even in that case, despite the high variability found at the taxonomic level, in the metabolic processes analysis, the result showed a clear overlapping of the processes. Although little described in aquaculture or animal production systems, this phenomenon is very constant and well characterized in ecology, as it has been widely investigated in relation to many ecosystems and environments, such as soil, sediments, and water, but also associated with organisms including humans [46,88,89,90,91,92]. It should be acknowledged, however, that the functional profiles presented here are based on 16S rRNA gene inference. Thus, while this approach allowed for valid cross-study comparisons between species, the predicted pathways should be interpreted as indicative of functional potential rather than direct evidence of gene content or activity. Future validation through metagenomics or metatranscriptomics, expanding the number of analyzed samples, will be necessary to provide more accurate insights and strengthen the conclusions regarding these metabolic signatures.

Parallel to the genetic differences within a single species, evolutionary distance between fish species also reflects cumulative genetic divergence and environmental adaptations that may shape microbiota [93]. Closely related species that share ecological niches or habitats often present more similar microbial communities than distant ones, suggesting that host phylogeny also plays a key role in structuring gut microbiota across taxa [94,95]. In the present study, to investigate this aspect, the two intestinal microbiota datasets of sea bass and trout were compared with a previously obtained sea bream gut microbial profile. In accordance with the premises, the PLS-DA results have shown that the adaptation to the marine environment and probably the smaller evolutionary distance determined a higher degree of similarity between the two saltwater fish species, separating them from the rainbow trout, which instead preferentially lives in fresh water and belongs to a more ancient taxonomic order, distant from the Perciformes [96,97]. Furthermore, these findings acquire greater importance because they show the same trend at multiple levels. This similarity is clear not only considering the total microbiota population, including transient and environmentally acquired microbes, but also in the core microbiota fraction, which mainly reflect host-specific long-term microbe associations [78,93]. Given this emerging tendency, such an approach, even considering the limited number of species used in this work, suggests a dichotomy which separates the two different ecological phenotypes. Further and broader studies are required to validate and refine this pattern; however, as a starting point, our findings highlight specific taxa which most influenced this separation. Especially relevant is that these discriminant bacteria belong almost entirely to the two core microbiota fractions obtained, indicating that, even in a convergent functional situation, the core bacteria are the main actors in the hierarchy of the entire population considering both their abundance and their functional contribution, as metabolically active and integrated into the physiology of the combined microbiota–host system (holobiont). Numerous authors, in fact, identified how these microbial markers were receptive to changes in different factors, especially in different dietary sources. The family Mycoplasmataceae, and in particular the genus *Mycoplasma*, are the taxa mainly reported when describing the salmonids’ intestinal microbiota due to their great abundance; however, other bacteria were also detected in the core bacterial populations of rainbow trout [98,99]. These genera include *Clostridium*, *Deefgea*, and *Shewanella* and different species of the Lactobacillaceae family. In the same way, bacteria like *Vibrio*, *Streptococcus*, *Pseudomonas*, *Micrococcus*, *Acinetobacter*, and *Paracoccus* not only usually represent the consistently abundant part of the intestinal populations of sea bream and sea bass but are also considered key contributors to the functional synergy within the fish gut ecosystem of these farmed species [17,100,101,102].

## 5. Conclusions

This study highlights the relevance of combining a taxonomic-functional perspective with computational network approach to unravel the hierarchy and the cooperation within the intestinal microbiota structure of farmed fish. In the present context of high intra-species taxonomic variability and strong functional redundancy, driven by factors like diet, environment, and genetics, our results showed how the definition and evaluation of the core microbiota represent a useful approach for defining both host-specific and general microbial features, acting as feasible synthesis of the entire bacterial profile. The application of Bayesian network modeling also allowed us to deepen the role of the core as a nerve center of interaction through the identification of closely related taxa that also act as keystone nodes in the structure–function hierarchy within the community. Thus, the association between core microbiota and specific potential microbial biomarkers form part of the transition in the use of microbiota from a descriptive variable into a strategic resource for sustainable aquaculture development. The integration of a multi-species perspective, moreover, adds a further dimension to the present microbiota study. The investigation of the convergent ecological pressures and habitat adaptation (freshwater vs. saltwater) of different farmed species allowed for the distinction of conserved and effective microbial traits, not only based on presence or abundance but also contemplating their ecological roles, relationships, and functional contributions. Ultimately, these exploratory starting results, together with those that will be obtained from the repetitive application of this methodological framework and more in-depth metagenomics and metatranscriptomics approaches, will define the list of taxa or functional clusters that in standard conditions constitute the species/eco-phenotype healthy microbial reference. This ambitious goal aims to use this novel information to design next-generation probiotics and prebiotics and as a guide to improve the fine-tuning of farming conditions, functional feed formulations, or the application of microbiota transplants, in order to promote fish growth performance, immunity, and welfare in aquaculture.

## Data Availability

All the microbiota datasets used in this study can be found online associated with their respective publications [18,48,49,50,51,52,53,54].

## Supplementary Materials

The following supporting information can be downloaded at: https://www.mdpi.com/article/10.3390/ani15192885/s1. Figure S1: Graphical representation of the validation of the PLS-DA model by the random permutations shown in Figure 2. Representation of the contribution of each component to variance explained (R2Y) and predicted (Q2), driving the separation of the four trials considered for sea bass (**a**) and the three trials considered for trout (**b**); Figure S2: graphical representation of the validation of the PLS-DA model by the random permutations shown in Figure 7. Representation of the contribution of each component to variance explained (R2Y) and predicted (Q2), driving the separation of the fish species considered (sea bass, sea bream, and trout) (**a**) and the two ecological grouping (freshwater and saltwater) (**b**). Table S1: Table summarizing dietary information during feeding trials for both sea bass and trout. Table S2: Summary of the statistical differences (*p*-value obtained by Kruskal–Wallis test) between sea bass and trout values of the inferred metagenomic functional enrichment analysis. The results are reported for both whole populations and core microbiota and divided by level 2 and level 3 of KEGG pathway description assignations. Table S3: Centrality metrics of the nodes composing the two Bayesian networks (sea bass and trout), including centrality degree, betweenness, and closeness.

## Abbreviations

The following abbreviations are used in this manuscript:

BN	Bayesian network
FM	Fishmeal
FO	Fish oil
OTUs	Operational taxonomic units
ZINB	Zero inflated negative binomial
BIC	Bayesian information criterion
MI	Mutual information
KEGG	Kyoto Encyclopedia of Genes and Genomes
PLS-DA	Partial least squares discriminant analysis
VIP	Variable importance in projection
