# Supplementary figures and images for: One Function, Many Faces: Functional Convergence in the Gut Microbiomes of European Marine and Freshwater Fish Unveiled by Bayesian Network Meta-Analysis
Source: Animals (Basel). 2025 Oct 2;15(19):2885. doi: 10.3390/ani15192885 (PMC12524309; doi:10.3390/ani15192885)

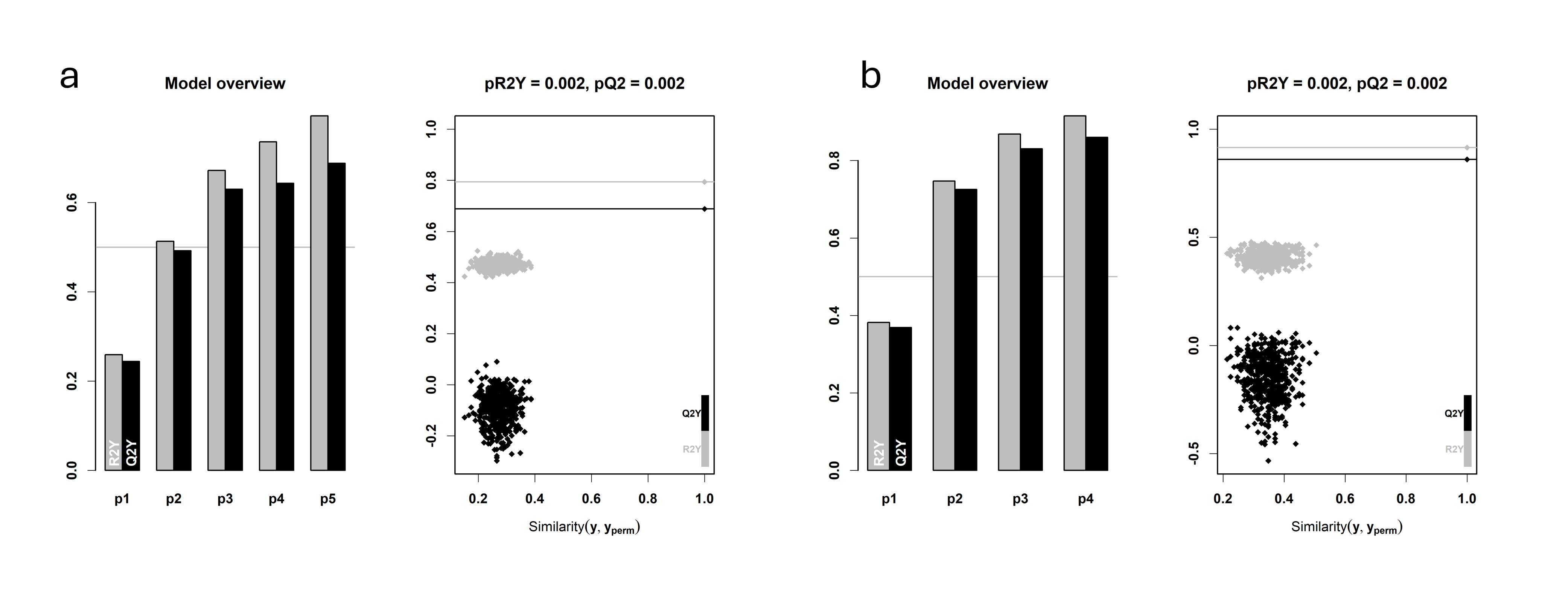

Supplement: Supplementary file 1 [file animals-15-02885-s001.zip › Figure S1.tif]

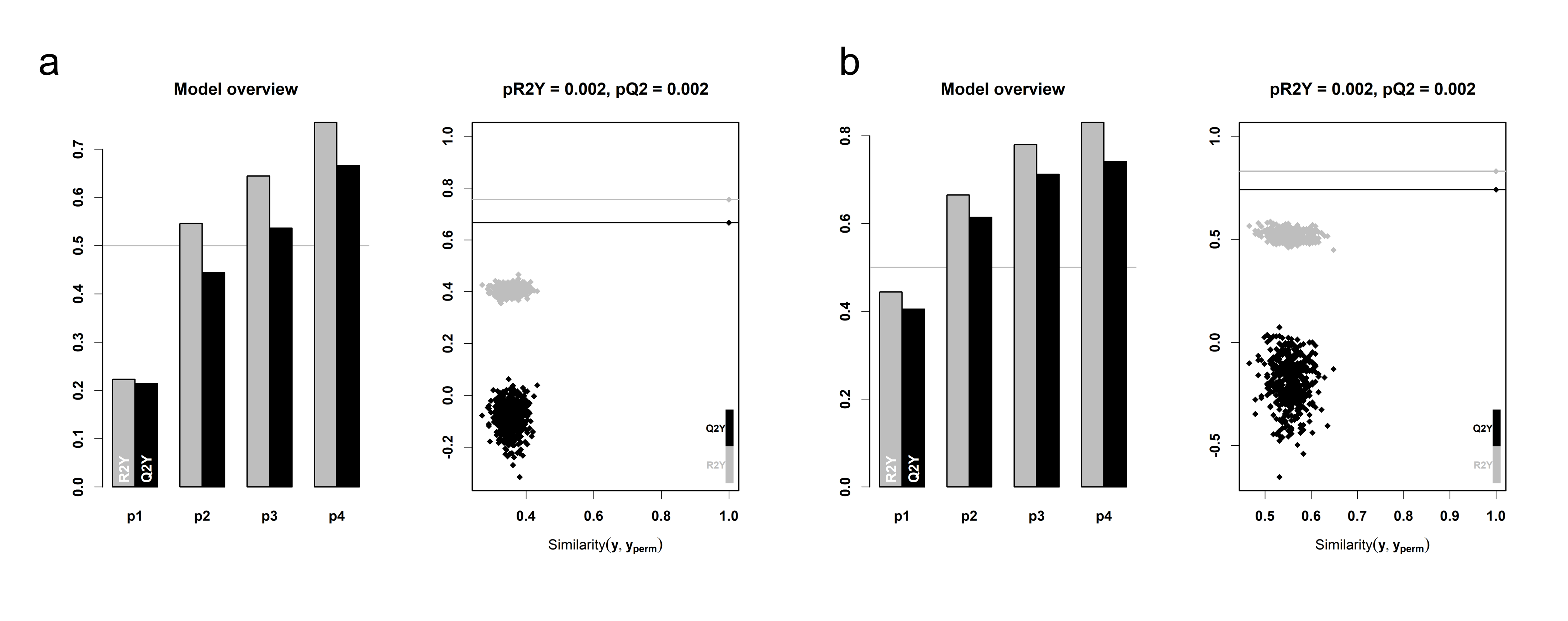

Supplement: Supplementary file 1 [file animals-15-02885-s001.zip › Figure S2.tif]
